# Ganciclovir-Resistant Cytomegalovirus Infection in a Kidney Transplant Recipient Successfully Treated with Foscarnet and Everolimus

**DOI:** 10.1155/2016/2736805

**Published:** 2016-01-31

**Authors:** Viola Menghi, Giorgia Comai, Olga Baraldi, Giovanni Liviano D'Arcangelo, Tiziana Lazzarotto, Gaetano La Manna

**Affiliations:** ^1^Department of Experimental, Diagnostic and Specialty Medicine, Nephrology, Dialysis and Transplantation Unit, St. Orsola Hospital, University of Bologna, 40138 Bologna, Italy; ^2^Clinical Microbiology Unit, St. Orsola Hospital, University of Bologna, 40138 Bologna, Italy

## Abstract

Cytomegalovirus (CMV) infection remains a major cause of morbidity, graft failure, and death in kidney transplant recipients. We describe a case of a 53-year-old CMV-seronegative man who underwent renal transplant from a CMV-positive donor and who developed ganciclovir- (GCV-) resistant CMV infection. Foscarnet was started while immunosuppressive therapy was modified with the introduction of everolimus minimizing tacrolimus dosage. Only two weeks after the start of this treatment regimen was the patient's viral load negative. At two-year follow-up the patient has no clinical or laboratory signs of CMV infection and a good and stable renal function or graft survival. In our case, administration of an mTOR inhibitor combined with foscarnet led to rapid and persistent viral clearance without compromising short- and medium-term graft function. This combination therapy supports the need for the kidney transplant community to individualize a target therapy for each type of GCV-resistant CMV infection.

## 1. Introduction

The direct and indirect effects of cytomegalovirus (CMV) infection in kidney transplant recipients make it a leading cause of morbidity, graft loss, and sometimes death [1, 2].

CMV infection can be primary in an IgG-CMV-seronegative patient with an IgG-CMV-positive donor or stem from viral reactivation. Early diagnosis is essential based on identification of viral replication by pp65 antigenemia in peripheral blood leukocytes or CMV DNA detection by PCR in blood or other biological tissues [3].

Ganciclovir (GCV) and valganciclovir (VGCV) currently represent the first-line antiviral drugs for the prevention and treatment of CMV infection. Nonetheless, a number of factors, including sustained GCV levels for prolonged periods, lack of specific CMV immunity, type of graft, potent immunosuppression, suboptimal antiviral drug levels, and delayed viral clearance during treatment, have contributed to drug resistance as an emerging therapeutic problem [4]. Prophylaxis is obviously recommended in CMV-seronegative transplant recipients from a seropositive donor, but it is associated with a theoretically increased risk of drug resistance. Couzi et al. [5] demonstrated that initial viral load may also be responsible for a high incidence of drug resistance. The clinical presentation of GCV-resistant CMV infection varies from asymptomatic forms [6] to organ dysfunction [7].

We report a case of a kidney transplant recipient with GCV-resistant CMV infection due to a mutation of the UL97 gene at codon C603W. The patient received a combination therapy with everolimus and foscarnet leading to a favorable outcome in terms of viral clearance and graft function.

## 2. Case Presentation

A 53-year-old CMV-seronegative man with hepatorenal polycystic disease who had been on hemodialysis for seven years underwent kidney transplant from a CMV-seropositive deceased donor in 2012.

The transplant was carried out without any surgical, immunological, or infectious complications. Immunosuppression was based on basiliximab induction therapy, followed by IV and then per os steroids tapered to a maintenance dose (oral prednisone 5 mg/die), tacrolimus (0.2 mg/kg/die to achieve trough level of 8–10 ng/mL for the first month and 5–8 ng/mL thereafter), and mycophenolic acid (720 mg/die). In view of the CMV R+/D− serological mismatch, the patient was given oral VGCV prophylaxis (900 mg/die based on renal function) for six months.

CMV pp65 antigenemia monitoring was negative during the patient's hospital stay and on discharge. He was discharged two weeks after transplant with serum creatinine up, 1.8 mg/dL. Serial outpatient follow-up showed a good kidney graft trend with serum creatinine up to 1.6 mg/dL at the one-month follow-up visit after transplant surgery. Laboratory tests 40 days after transplantation showed a serum creatinine increase to 1.9 mg/dL associated with raised blood immunosuppression levels (tacrolimus: 19.3 ng/mL). The patient reported diarrhea for several days. pp65 antigenemia monitoring now showed >300 cells and quantitative PCR assay of the viral genome showed 164,440 copies/mL.

The patient was admitted to hospital for further tests and treatment. On admission his general clinical status was good with normal vital parameters, no fever, and no diarrhea.

Chest X-ray and esophagogastroduodenoscopy (EGD) were negative. Biohumoral tests were normal except for serum creatinine, 2 mg/dL. Oral VGCV was suspended and IV GCV (5 mg/kg/12 h) initiated. Immunosuppression was adjusted with suspension of mycophenolic acid, also in light of his recent bowel symptoms, combined with reduced calcineurin inhibitor dose (to achieve tacrolimus trough level of 5 ng/mL).

Despite antiviral therapy, there was no reduction in CMV DNAemia. Ten days after the start of treatment, genetic screening for suspected drug resistance disclosed a mutation of the UL97 gene at codon C603W (substitution of cysteine for tryptophan) conferring resistance to VGCV and GCV [8]. Based on the algorithm recently confirmed in the Guidelines of the Transplantation Society [9], GCV was suspended and antiviral therapy switched to foscarnet with dosage adjusted for the patient's weight and kidney function (6 g/die).

Foscarnet was infused at a concentration of 24 mg/mL with dilution prior to administration through a jugular venous catheter.

In light of several literature reports on the efficacy of mTOR inhibitors in GCV-resistant CMV infection [10] our patient's immunosuppressive regimen was modified with the introduction of everolimus (trough level of 3–8 ng/mL) and a further reduction of tacrolimus dosage (trough level of 1–3 ng/mL). Mycophenolic acid was not used more.

Kidney function, blood count, and serum and urine electrolytes were monitored throughout treatment. Viral load was determined by PCR every three days.

Combination therapy with foscarnet and everolimus did not worsen kidney function. The patient presented three episodes of mild fever (up to 37.5°C) that resolved spontaneously. Calcium and magnesium supplementation were administered for transient hypocalcemia (7.5 mEq/L; range 9–10.7 mg/dL) and hypomagnesemia (1.1 mEq/L; range 1.4–1.85) with good response.

**Figure 1:**
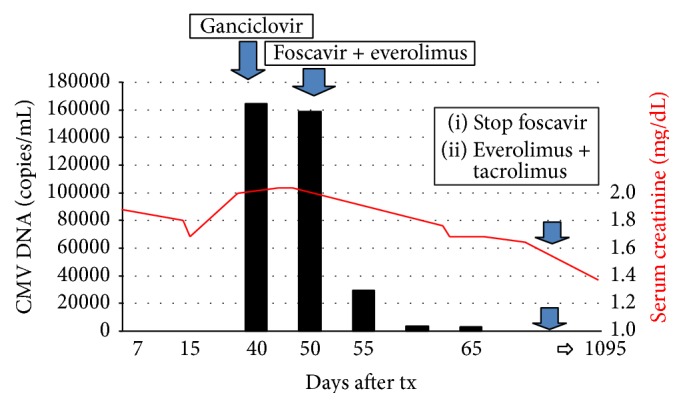
Laboratory trend of serum creatinine levels (line) and CMV DNA on plasma (bars) over time and therapy administered. Ten days after the start of GCV therapy genetic screening disclosed a mutation of the CMV UL97 gene at codon C603W. GCV was suspended and antiviral therapy switched to foscarnet with dosage adjusted for the patient's weight and kidney function (6 g/die). At the same time the immunosuppressive regimen was modified with the introduction of everolimus, minimizing tacrolimus dosage. Two weeks after initiating this new therapeutic approach, the patient presented a clearance of viral load with no repercussions for kidney function. This trend persisted at three years after transplantation with serum creatinine level of 1.3 mg/dL.

Two weeks after the start of foscarnet and everolimus combination therapy, the patient presented complete viral clearance (negative PCR) so foscarnet was suspended maintaining the immunosuppression regimen with everolimus (trough level of 3–8 ng/mL) and tacrolimus (trough level of 1–3 ng/mL). The patient was discharged with a serum creatinine level of 1.6 mg/dL. Subsequent PCR assays for CMV remained negative (<500 copies) and kidney function was stable (Figure 1). The patient was monitored by DNA PCR testing every three months for two years. One year after transplant kidney function was 1.6 mg/dL with negative DNAemia.

One-year protocol kidney allograft biopsy disclosed no signs of CMV infection or graft rejection but only aspecific mild focal chronic parenchymal changes (Banff 2013 score: acute alterations: all negative; chronic alterations: interstitial fibrosis 2+, tubular atrophy 1+, arterial fibrointimal thickening, negative transplant glomerulopathy, and arteriolar hyalinosis; negative peritubular capillaries C4d deposition; CMV and BKV nephropathy, both negative). At three-year follow-up the patient's serum creatinine level is 1.3 mg/dL with persistently negative CMV infection. His immunosuppressive regimen remains unchanged.

## 3. Discussion

Ganciclovir (GCV) resistance is an emerging therapeutic challenge and should be suspected in patients failing to show a clinical and virological response to prolonged therapy. Diagnosis of GCV-resistant CMV infection is established by demonstration of mutations in the UL97 kinase or UL54 DNA polymerase genes with analysis of CMV DNA [9].

More than 90% of cases presenting GCV resistance are caused by a mutation of the UL97 gene encoding a kinase and phosphorylase protein in the GCV site. Once activated, GCV inhibits viral DNA polymerase. The UL97 mutation impairs GCV phosphorylation thereby preventing the drug's action in infected cells. In particular, the mutations at codons 460, 520, 594, 595, 599, 603, and 607, along with deletions of codons 590–593, all confer GCV resistance on laboratory CMV strains, usually resulting in a 5- to 10-fold elevation of IC_50_ values [11].

No controlled trials currently support a best practice for the selection of an alternative treatment in cases of GCV-resistant CMV infection. However, the International Consensus Guidelines of the Transplantation Society advocate a diagnostic-therapeutic algorithm. Firstly, when GCV resistance is suspected it is important to rule out subtherapeutic drug levels and excessive immunosuppressive therapy. If a UL97 gene mutation is disclosed in certain sites, as in our case at codon C603W, the algorithm suggests switching to foscarnet.

Little information is currently available on the clinical use of mTOR inhibitors in the treatment of GCV-resistant CMV infection.

A few recent studies have demonstrated the potential benefit of leflunomide in refractory cases for its both antiviral and immunosuppressive properties, but its effective benefits are limited to a small cohort of treated patients [12].

We describe a successful therapeutic approach with foscarnet and everolimus in a kidney transplant recipient with a GCV-resistant infection.

As regards the use of mycophenolic acid, we preferred to stop this type of immunosuppressant directly both for the patient's clinical symptoms (diarrhea) and particularly for the evidence of several studies showing that this drug is associated with an increased risk of CMV disease [13, 14].

However, other studies suggest that it is the degree of immunosuppression that determinates the increased risk of CMV infection and not the drug itself [15, 16].

The limit of this presentation is the lack of a case control treated with foscarnet but not everolimus, so we can not determine the role that everolimus was playing.

A recent study by Myhre et al. [17] evaluated the incidence, type of genetic mutation, and outcome in a sample of 1244 kidney transplant recipients over an observation period of five years. They found that two subjects presented the mutation at codon C603W, the same as in our patient, with plasma viral load 3,485 and 19,600 copies/mL. One of the GCV-resistant patients in Myhre et al.'s study was treated with foscarnet with a viral clearance time of 39 days; the other patient received VGCV and took 209 days to achieve viral clearance.

At the time of GCV-resistant CMV diagnosis, our patient had a higher viral load (164,400 copies/mL). We adopted a hybrid strategy in our patient based on the efficacy of immunosuppression with mTOR inhibitors on outcome in terms of eradicating GCV-resistant infection of recent studies [10] and on the latest guidelines recommending foscarnet administration [9].

The combined treatment adopted in our patient led to rapid viral clearance after just two weeks. The therapy was well tolerated with an excellent outcome in terms of both kidney function and medium-term graft function.
